# Emerging nanoprobes for the features visualization of vulnerable atherosclerotic plaques

**DOI:** 10.1002/SMMD.20240033

**Published:** 2024-12-03

**Authors:** Xin Wang, Dan Mu, Jing Liang, Ruijing Xin, Yukun Zhang, Renyuan Liu, Mei Yao, Bing Zhang

**Affiliations:** ^1^ Department of Radiology The Affiliated Drum Tower Hospital of Nanjing University Medical School Nanjing China; ^2^ Medical Imaging Center Affiliated Drum Tower Hospital Medical School of Nanjing University Nanjing China; ^3^ Institute of Medical Imaging and Artificial Intelligence Nanjing University Nanjing China; ^4^ Department of Radiology Drum Tower Hospital Clinical College of Nanjing Medical University Nanjing China; ^5^ Jiangsu Key Laboratory of Molecular Medicine Nanjing China; ^6^ Institute of Brain Science Nanjing University Nanjing China

**Keywords:** molecular imaging, nanoprobes, pathological features, visualization, vulnerable atherosclerotic plaques

## Abstract

Atherosclerosis (AS) is a major cause of cardiovascular disease. In particular, the unpredictable rupture of vulnerable atherosclerotic plaques (VASPs) can cause serious cardiovascular events such as myocardial infarction, stroke, and even sudden death. Therefore, early evaluation of the vulnerability of atherosclerotic plaques is of great importance. However, clinical imaging techniques are only marginally useful in the presence of severe anatomical structural changes, making it difficult to evaluate plaque vulnerability at an early stage. With the development of molecular imaging and nanotechnology, specific nanoprobes constructed for the pathological features of VASPs have attracted much attention for their ability to visualize VASPs early and noninvasively at the cellular and molecular levels. Here, we outline the pathological features of VASPs, analyze the superiority and limitations of current clinical imaging techniques, introduce the rational design principles of nanoprobes, and systematically summarize the application of nanoprobes to visualize the features of VASPs at the cellular and molecular levels. In addition, we discussed the prospects and urgent challenges in this field, and we believe it will provide new ideas for the early and accurate diagnosis of cardiovascular diseases.

## Graphical abstract

The pathophysiology of atherosclerosis and the rational design principle of nanoprobes for the feature visualization of vulnerable atherosclerotic plaques are introduced. In addition, the nanoprobes for the feature visualization of vulnerable atherosclerotic plaques are discussed.

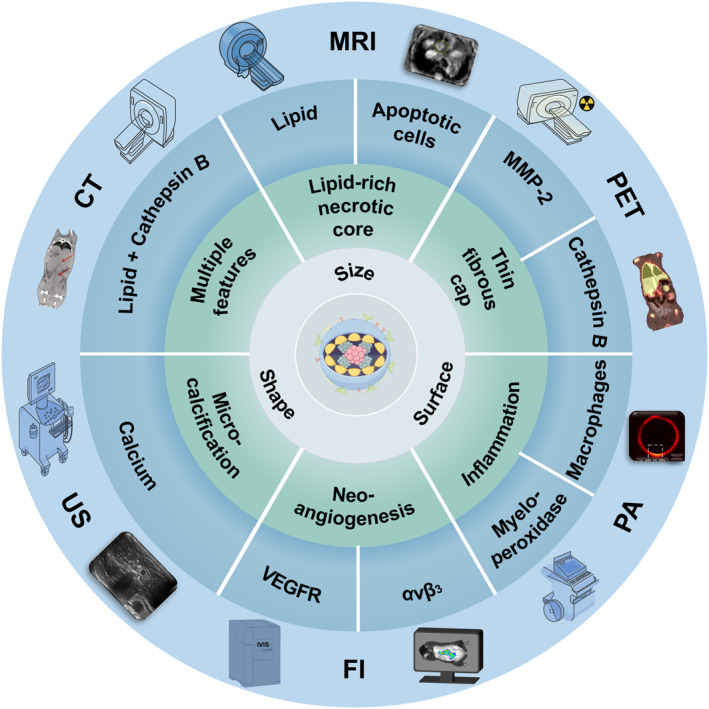

**Key points**

- The rational design principle of nanoprobes for the feature visualization of vulnerable atherosclerotic plaques is introduced.
- The nanoprobes for the feature visualization of vulnerable atherosclerotic plaques are discussed.
- Current trends and future directions of nanoprobes in the diagnostic of vulnerable atherosclerotic plaques are prospected.

## 1 INTRODUCTION

With the rapid development of social economy and the aggravation of people's living pressure, cardiovascular disease (CVD) has gradually become one of the major chronic diseases and causes of death in the world, accounting for 32% of global deaths.^1^ Atherosclerosis (AS), which was considered as a chronic inflammatory disease initiated by the dysfunctional endothelial cells and deleterious accumulation of lipids, remains the leading cause of cardiovascular diseases (CVDs).^2^ Especially, the sudden rupture or superficial erosion of vulnerable atherosclerotic plaques (VASPs) triggers some fatal cardiovascular events such as myocardial infarction, stroke, and even sudden cardiac death.^3^, ^4^ The main obstacle to overcoming VASPs is their asymptomatic progression before a serious vascular occlusion or a traumatic event caused by plaque rupture. Therefore, early screening is extremely important for the treatment of VASPs.

Currently, the most used clinical strategies for screening VASPs are the medical imaging technologies, which are mainly divided into non‐invasive and invasive techniques. Magnetic resonance imaging (MRI), ultrasonography (US) and X‐ray computed tomography (CT) were applied for non‐invasive diagnosis of VASPs. For example, CT was employed for calcium scoring of vascular.^5^ MRI was used for detecting the plaque components including lipid‐rich necrotic core and intraplaque hemorrhage due to its super spatial resolution.^6^ Ultrasound was introduced for assessing the degree of plaque as intima‐media bulge, total plaque area/volume and blood flow.^7^ Invasive techniques such as intravascular ultrasound (IUVS) and optical coherence tomography (OCT) were applied for measuring the plaque components and detailed morphological information, respectively.^8^ Disappointedly, these imaging strategies could only image AS with a marked structure but cannot reveal information about the pathological features and biological compositions within the plaques, which is more responsible for plaque rupture, leaving serious deficiencies in assessing plaque vulnerability at the early stage of AS.^9^ Thus, much work remains to be explored in developing new strategies for visualizing VASPs at the cellular and molecular levels.

Benefitting from the developments of molecular imaging, early VASPs visualization has made great progresses.^10^, ^11^, ^12^ Molecular imaging is a kind of medical imaging that provides a detailed picture reflecting what is occurring in the body at the cellular and molecular level. Because biochemical changes precede anatomical changes, molecular imaging holds huge promise for early visualization of diseases. Most molecular imaging strategies require the use of imaging contrasts, usually intravenously, that interact with the target environment to reveal biological pathways.^13^ Therefore, designing imaging contrasts with good biocompatibility, high binding affinity and specificity is urgently needed but challenging. In recent years, the rapid development of nanotechnology has promoted the progress of molecular imaging.^14^, ^15^, ^16^ Nanoprobes exhibit significant advantages in extending systemic circulation time due to their unique size effect, allowing them to accumulate more at lesion sites via the enhanced permeability and retention (EPR) effect similar with that in solid tumors.^17^ In addition, nanoprobes, with large surface areas, could be easily modified according to the pathological features, to perform diverse functions such as lesion‐targeting, specificity, achieving smart control and improving diagnostic sensitivity and specificity.^18^ An in‐depth understanding of the features of VASPs promises new ideas for constructing novel targeted nanoprobes.

The development of AS is a complex process involving multiple cells and molecules, resulting in the clinical features of VASPs including: (1) lipid‐rich necrotic core; (2) thin fibrous cap; (3) inflammation; (4) neo‐angiogenesis; (5) microcalcification. The biomarkers related to these features mainly involve lipids, apoptotic cells, protease, inflammatory macrophages, integrin (αvβ_3_), vascular endothelial growth factor (VEGF) and calcium.^19^ For example, deposition of lipids in the intima induces an inflammatory response that recruits inflammatory cells to overload phagocytosis of the lipids, leading to apoptosis. Lipids, together with the apoptotic cells, constitute a major component of the lipid‐rich necrotic core. In recent years, there has been a proliferation of targeted nanoprobes based on these biomarkers to improve the sensitivity and specificity of VASPs visualization.^20^, ^21^, ^22^ In particular, the nanoprobes that recognize multiple features simultaneously could improve the accuracy of VASPs detection.

**SCHEME 1:**
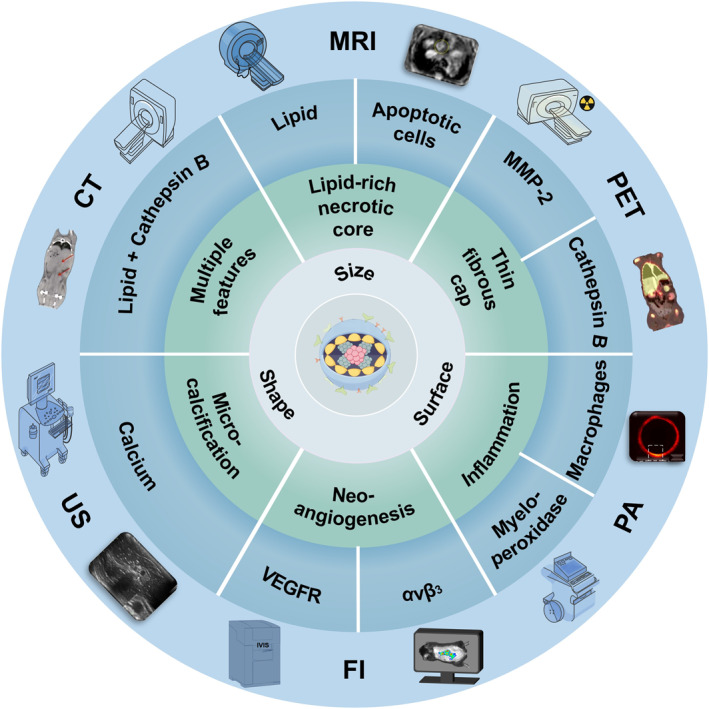
A schematic overview of nanoprobes for the feature visualization of vulnerable atherosclerotic plaques.

As a result, nanoprobes for visualizing the features of VASPs bring a new era to the early detection of VASPs. So far, there are many excellent reviews summarizing the ability of nanomaterials for screening VASPs.^23^, ^24^, ^25^, ^26^, ^27^, ^28^ However, to the best of our knowledge, there is no comprehensive and systematic review of nanoprobes for specific visualization of VASPs from the perspective of features. Herein, we focus on the cutting‐edge research of nanoprobes used for early visualization of VASPs features. As shown in Scheme 1, firstly, we outlined the pathophysiology of AS and conducted the features of VASPs. Secondly, we summarized the mechanisms, superiorities, and limitations of commonly used clinical imaging strategies. Thirdly, we introduce the principle of rational design of nanoprobes. Fourthly, we highlight the state‐of‐the‐art nanoprobes for visualizing the features of VASPs. Then, we discussed the current prospects and challenges in the fields of early visualization of VASPs based on nanoprobes.

## 2 THE PATHOPHYSIOLOGY OF AS

**FIGURE 1:**
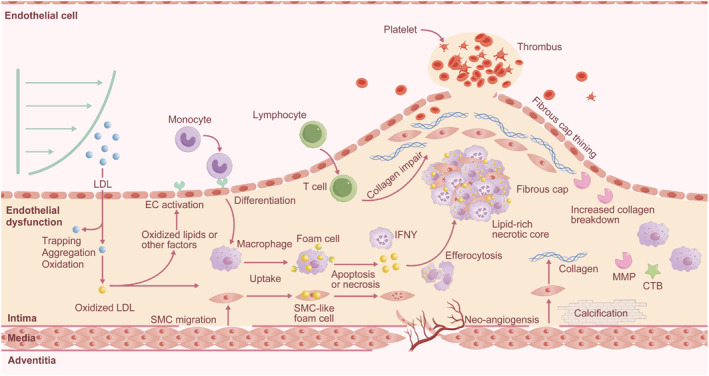
Schematic illustration of the development of atherosclerosis.

AS is considered as a chronic disease driven by inflammation. The pathogenesis of AS was conveniently categorized into three stages: initiation, progression, and complication,^29^ as shown in Figure 1.

### 2.1 Initiation

There are three fundamental processes involved in the initiation of atherosclerosis: low‐density lipoprotein (LDL) deposition, endothelial dysfunction and inflammation. The normal artery wall consists of a trilaminar structure: the adventitia (the outermost layer), media, and intima (the innermost layer). Due to the impaired endothelium caused by some cardiovascular risk factors such as smoking, hypertension or abnormal blood flow shear stress, low‐density lipoprotein (LDL) deposits in the intima, loses the protection of antioxidants in plasma, and undergoes oxidation and other modifications, resulting in pro‐inflammatory and immunogenicity features.^30^ In response to this situation, endothelial cells that line the intima express adhesion molecules for recruiting and attaching the monocytes circulating in the blood.^31^ Then, the bound monocytes migrate to the artery wall under the stimulation of chemokines. Once inside the intima, monocytes mature into macrophages which bind and engulf the oxidized LDL particles under the recognition of surface scavenger receptors. When macrophage cells fail to fully dispose the lipids, they become lipid‐laden foam cells.^32^ A recent study has shown that smooth muscle cells (SMCs) can also uptake lipid to form foam cells in common with macrophages.^33^ These inflammatory cells secrete proinflammatory factors, reactive oxygen species (ROS) and tissue factor coagulants thereby exacerbating local inflammation and increasing the occurrence of thrombotic complications.^34^

### 2.2 Progression

AS proceeds through the continuous accumulation of lipids and lipid‐laden cells. With excessive uptake of foam cells to cholesterol, its lipid metabolism becomes overwhelmed, leading to cell apoptosis and further release of lipids. Defective clearance apoptotic cells (efferocytosis) contribute to the accumulation of cellular debris, which together with lipids form the necrotic core.^35^ The resident and recruited SMCs play an important role in thickening the plaque. In the intimal region, the SMCs proliferate under the regulation of platelet‐derived growth factor and produce extracellular matrix molecules (ECMs) including interstitial collagen and elastin to form a fibrous cap on the plaque.^36^ However, the activated macrophages will secrete matrix metalloproteinases (MMPs) family to degrade the interstitial collagen, resulting in the weakening of the fibrous cap and increasing the susceptibility of plaque rupture.^37^ In addition, the activated macrophages also increase the production of procoagulant tissue factors that trigger the formation of thrombus.^38^ Although less abundant than macrophages, T cells also regulate the plaque evolution in many aspects.^39^ For example, T_H_1 cells produce IFNγ that can impair the ability of the SMCs to synthesize interstitial collagen, thereby promoting AS, but T_H_2 and T_reg_ cells can ameliorate inflammation. Growing atherosclerotic plaques increase the demand for oxygen and nutrients in the plaque due to the accumulation of lipids and inflammatory cells on the one hand, and on the other hand, plaque growth leads to localized hypoxia which in turn produces hypoxia‐inducible factors that modulate neo‐angiogenesis.^40^ During their evolution, many plaques will be calcified to a certain extent in response to inflammation. Microscopic or spotty calcification will promote the plaque mechanically unstable and prone to rupture.^41^

### 2.3 Complication

With time, the necrotic core becomes larger and the inflammation becomes more serious, which makes the fibrous cap thinner and finally leads to the rupture of plaque. The rupture of atherosclerotic plaque will expose the thrombogenic substances in the necrotic nucleus, especially the tissue factors produced by macrophages, to the blood cavity, thus leading to the formation of thrombus, which is the ultimate and most terrible complication of atherosclerosis. Occlusive thrombosis, together with the impairment of local dynamic balance function of vascular endothelial cells, can cause ischemic damage, such as stroke and acute coronary syndrome. Thrombosis may also lead to severe limb ischemia and complicate peripheral arterial disease.^42^

Numerous autopsy histopathologic studies have identified the key features of VASPs, including (1) lipid‐rich necrotic core; (2) thin fibrous cap; (3) inflammation; (4) neo‐angiogenesis; (5) microcalcification.^43^, ^44^, ^45^ The lipid‐rich necrotic core and thin fibrous cap are typical features of VASPs. The growing necrotic core gradually breaks through the protection of the thinning fibrous cap, leading to plaque rupture. Inflammation occurs throughout the development of AS, recruiting inflammatory cells (mainly macrophages) that enlarge the necrotic core on the one hand, and on the other hand, inflammatory cells secrete proteases that thin the fibrous cap. Neo‐angiogenesis is unstable and prone to rupture, leading to intraplaque hemorrhage and increased inflammation. Microcalcifications can increase the volume of the necrotic core and increase the blood flow shear stress on the plaque, leading to plaque rupture. Clinical imaging techniques also often determine plaque vulnerability by characterizing these features. However, these structural features often do not appear until the plaque has progressed to an advanced stage, thus preventing early diagnosis. Molecular imaging based on specific biomarkers of these features allows for the early diagnosis of VASPs.

## 3 CURRENT CLINICAL IMAGING TECHNIQUES FOR AS

Up to now, in view of the features of the anatomical structure formed during the development of AS, there are many imaging technologies used in clinic, commonly divided into invasive and non‐invasive techniques. Imaging information can help clinicians grade the risk of future cardiovascular events. Here, we briefly introduce how these techniques assess plaque features, as well as their superiority and limitations (Table 1).

**TABLE 1:** Mechanism, features detected, advantages and disadvantages of clinical imaging techniques.

Techniques		Mechanism	Features detected	Advantages	Disadvantages
Invasive imaging techniques	CAG	Contrast agent is injected through a catheter and visualized under x‐ray irradiation	Plaque burden	Real‐time High resolution	Invasive Radioactive No wall imaging
	IVUS	A miniature ultrasound probe is placed through a catheter into the target vessel and imaged under the ultrasound	Large necrotic core Highly calcification	Real‐time Visualization of composition	Invasive and expensive Low resolution Limited in small vessels
	OCT	Similar with IVUS, except that light replaces ultrasound	Large necrotic core Highly calcification thin fibrous cap	Real‐time Visualization of composition High resolution	Invasive Need to block the blood Low penetrability
	NIRS	Similar with OCT, except longer wavelengths	Lipid content	High resolution	Invasive Non‐specific
Non‐invasive imaging techniques	US	It emits ultrasound waves into the body through an ultrasound probe and achieves tissue imaging by recording the echo signals	Plaque burden Intima thickness Blood flow velocity	Non‐invasive No radiation Low cost Real‐time	Low spatial resolution Two‐dimensional
	CTA	Iodine‐based contrast agent is injected and visualized under x‐ray irradiation	Calcification Stenosis degree	Non‐invasive High specificity Easy to operate	Low spatial resolution Radioactive Limited in iodine allergy
	MRA	Collects the relaxation signal of hydrogen protons (^1^H) in water molecules under the action of a high magnetic field	Necrotic core Hemorrhage Plaque calcification Thin fibrous cap	Non‐invasive No radiation High spatial resolution	Low sensitivity Long imaging time Metal implants are limited
	PET	Detecting the intensity and distribution of injected radiolabeled tracers capable of binding to some specific molecules associated with certain biochemical processes.	Inflammation Micro‐calcification Neo‐angiogenesis	High sensitivity Quantifiable	Low spatial resolution Invasive Expensive Challenging for imaging of coronary arteries

### 3.1 Invasive imaging techniques

Invasive imaging techniques are used for intravascular imaging with the superiority of capturing images near the plaque, thus providing a very high‐resolution image of the anatomical structure.

#### 3.1.1 Coronary angiography

Coronary angiography (CAG) is a commonly used and effective imaging technique in which a special cardiac catheter is inserted into the coronary artery through the femoral or radial arteries, and then a contrast agent is injected to visualize the coronary artery under the X‐ray irradiation.^46^ It can clearly display the lumen of the entire coronary trunk and its branches, observe whether there are stenotic lesions in the blood vessels, and make a clear diagnosis of the location, extent, severity, and blood vessel walls of the lesions through a real‐time assessment of the flow of contrast agents in the coronary arteries.^47^ However, coronary angiography has its major drawbacks, such as its invasiveness and radiation. In addition, coronary angiography is difficult to identify diffuse stenosis because of its inability to image the vessel wall. Moreover, it may underestimate plaque burden since the walls of the vessels in patients with atherosclerotic coronary heart disease often dilate outward.^48^

#### 3.1.2 Intravascular ultrasound

Intravascular ultrasound (IVUS) is a medical imaging technology that combines non‐invasive ultrasound technology with invasive catheter technology. It places miniature ultrasound probes into the vessel of interest through cardiac catheters to directly observe the atherosclerotic plaque.^49^ Generally, due to the different acoustic impedance of the three‐layer structure of the vascular wall, the coronary artery shows alternating bright and dark areas on IVUS. However, atherosclerotic vessels have different appearances depending on the composition of the plaque. Highly calcified plaques will exhibit bright sound shadows while lipid rich plaques will have clearer echoes.^50^ Although IVUS provides a useful tool for identifying vulnerable coronary plaque lesions, there are some limitations to overcome when used to image AS. First, the resolution of IVUS is limited, so it is impossible to accurately measure the fibrous cap thickness. Second, due to the limitation of the size of the ultrasonic probe, IVUS is difficult to apply to the detection of lesions with small lumen diameter or severe stenosis of blood vessels. Third, IVUS is currently unable to distinguish between hyperechoic plaque and acute thrombosis. Finally, because of its invasive and expensive, the current guidelines do not recommend it for routine screening.^51^

#### 3.1.3 Optical coherence tomography

Optical coherence tomography (OCT) is a medical tomographic imaging technology that integrates semiconductor lasers, optics, ultra‐sensitive detection, and computer image processing technology. Its imaging mechanism is similar to IVUS except that light replaces ultrasound.^52^ Ultrasound generates images through reflected echoes, whereas OCT uses about 1300 nm infrared light to reflect the microstructure inside biological tissues. Because the bandwidth and wave speed of infrared light are many orders of magnitude higher than that of medical ultrasound, the resolution of the image obtained by OCT is 10 times that of IVUS, making it the “gold standard” for diagnosing fibrous atherosclerotic plaque of thin cap.^53^ However, OCT also has certain limitations in plaque imaging. When OCT is performed, blood flow must be blocked with a balloon, which is easy to cause tissue ischemia and arrhythmia. In addition, the penetrability of OCT is only 1–2 mm, making it impossible to reliably detect the medial and adventitia of the blood vessel wall. Finally, because OCT is an invasive imaging technology, it is not suitable for routine screening.^54^

#### 3.1.4 Near‐infrared spectroscopy

Near‐infrared spectroscopy (NIRS) is a catheter‐based invasive imaging technology, similar in operation to IVUS and OCT imaging, that uses near‐infrared light with a wavelength of 800–2500 nm to project onto the atherosclerotic plaque on the blood vessel wall and presents the reflected signal in the form of a spectrum. Then, with the aid of computer, the lipid content in the atherosclerotic plaque can be measured to obtain the lipid core load index (LCBI).^55^ However, it can only provide a lipid image of the vessel surface and does not provide specific information on the plaque depth of the lipid core, often used in combination with IVUS in clinical practice.^56^

Overall, invasive imaging has better resolution and more accurate assessment. However, it also carries some risk, plus patient compliance is poorer, and it is not easy to use.

### 3.2 Non‐invasive imaging techniques

Compared with the damage caused by invasive imaging to the body, non‐invasive imaging technology is widely popular in clinical practice due to its simplicity and non‐invasiveness. Ultrasound, CT and magnetic resonance imaging (MRI) are the most commonly used clinical non‐invasive imaging techniques for the detection of AS.

#### 3.2.1 Ultrasound

Ultrasound imaging (US) based on B‐mode is the use of ultrasound to achieve diagnosis and is the second most commonly used imaging technique in clinical practice. It transmits ultrasonic waves into the human body through an ultrasonic probe and realizes tissue imaging by recording echo signals in terms of the difference in tissue reflection of ultrasonic waves, often used to quantify the degree of stenosis caused by atherosclerotic plaque and to measure the plaque burden, with the advantages of non‐invasive, easy to obtain, portable, low cost, real‐time monitoring, and no ionizing radiation.^57^ However, its insufficient spatial resolution makes it difficult to find tiny lesions. Also, since its images are two‐dimensional, it leads to inaccurate estimation of overall plaque size.^58^

Although the later Doppler ultrasound technology quantifies the stenosis by measuring the blood flow velocity, which overcomes these shortcomings to a certain extent, it still has many limitations in practical application; for example, it requires the ultrasound beam to be as parallel as possible to the blood flow direction, and incorrect angles can lead to misestimations of stenosis. Also, if the color gain is set very high, the degree of stenosis within the vessel will be underestimated. Finally, tortuous carotid arteries often lead to overestimation of stenosis.^59^

#### 3.2.2 Computed tomography angiography

Computed tomography angiography (CTA) is a non‐invasive vascular imaging technique based on CT scanning technology which can be used to display the vascular system of the whole body, especially for displaying vascular calcification. It only needs to inject iodine‐based contrast agent intravenously, and the lesions that need to be contrasted can be displayed under the irradiation of X‐ray.^60^ Compared with CAG, it is non‐invasive and easy to operate. Coronary arteries can now be imaged in greater detail, which is attributed to the shorter acquisition times of newer generations of CT scanners. Indeed, CTA has high accuracy in determining the severity of coronary stenosis, especially in patients with suspected cardiogenic chest pain. However, the limited soft‐tissue resolution of CTA makes it difficult to distinguish components such as fibers, lipids, and hemorrhage within the plaque. In addition, it is radioactive and requires the application of an iodine contrast agent, so it is not suitable for patients with iodine allergy and renal insufficiency.^61^

#### 3.2.3 Magnetic resonance angiography

Magnetic resonance angiography (MRA), which primarily collects the relaxation signal of hydrogen protons (^1^H) in water molecules under the action of a high magnetic field, is best suited for imaging large, stable vessels such as the carotid artery.^62^ Imaging differences of tissue at different image weightings (T_1_, T_2_, and proton density) endow MRA with excellent soft tissue contrast and high resolution, allowing it to discriminate not only vessel lumens but also vessel walls for accurate plaque measurements size and thickness.^63^ More importantly, it can also be used to investigate the constituents within VASPs, such as lipid‐rich necrotic core, intraplaque hemorrhage, calcification, and thin fibrous cap, without the assistance of any ionizing radiation, allowing MRA suitable for repeated longitudinal examination of chronic cardiovascular disease to follow the progression of plaque.^64^ Gadolinium‐based contrast agents are commonly used clinically to enhance the contrast of the fibrous cap of atherosclerotic plaques and to indicate plaque vascularity and inflammation.^65^ However, there are still some deficiencies in its clinical application. First, its low sensitivity makes it ineffective in diagnosing early‐stage lesions. Second, its long imaging time leads to motion artifacts near the heart making imaging of small vessels challenging.^66^ Third, due to the huge magnetic field attraction, it is not suitable for cardiovascular patients with metal implants in their body. Finally, the nephrotoxicity of gadolinium‐based contrast agent also limits its clinical application to some extent.^67^

#### 3.2.4 Positron emission tomography

Positron emission tomography (PET) is the only non‐invasive imaging technique that can display biomolecular metabolism, receptors, and neurotransmitter activities in vivo. Its mechanism of action is to detect cell activity by detecting the intensity and distribution of intravenously injected radiolabeled molecular ligands or tracers capable of binding to some specific molecules associated with certain biochemical processes such as inflammation, microcalcification, neo‐angiogenesis.^68^ The signal of PET comes from foreign radio‐labeled tracer (not present in the body), making it ultra‐sensitive and quantifiable. However, it has some limitations in the detection of atherosclerosis. First, the low spatial resolution of approximately 6 mm makes it difficult to detect vulnerable plaques in smaller vessels. Second, in coronary imaging, the PET signal is degraded due to the heart's voluntary respiratory motion. In addition, during the detection of coronary artery inflammation, tracers can also be taken up by cardiomyocytes in large amounts, making it difficult to distinguish between arterial inflammation and myocardial uptake. Third, it requires the involvement of radioactive material, which is harmful to the human body, resulting in the inability to repeat the detection in a short period of time. Finally, it is more expensive which prevents it from being widely used.^69^

Overall, imaging techniques, both invasive and noninvasive, are usually primarily concerned with general morphologic parameters of the vessel lumen and vessel wall. The actual association of these features with the degree of plaque vulnerability is only indirectly relevant as pathologic results cannot be obtained by biopsy of most vessels. In addition, invasive and noninvasive imaging have their own advantages and disadvantages (Table 1), and the choice of imaging method depends on the specific clinical needs and the patient's specific situation. In practice, a combination of imaging techniques is often used to obtain the most accurate diagnostic results, depending on the patient's specific situation and diagnostic needs.

## 4 THE ADVANTAGES OF NANOPROBES‐BASED IMAGING AND THEIR RATIONAL DESIGN

Current imaging techniques can only visualize structures that have undergone significant deformation, such as narrow lumens, large necrotic cores, and high calcification, so that early plaques cannot be detected in time. Moreover, these structural features obtained by current imaging strategies sometimes do not reflect plaque vulnerability. Advances in molecular imaging have promoted the early detection of plaques, especially in assessing plaque vulnerability. The research of molecular imaging probes is the biggest driving force for the development of molecular imaging.^70^

Advantages: Researchers have been working on designing new probes based on small‐molecules or peptides to visualize VASPs until the advent of nanoprobes. Although small molecules can easily spread in the body and often enter cells, unlike nanoprobes, they cannot host numerous payloads, cannot usually be combined with therapies, and cannot be accurately or easily engineered. Furthermore, small molecules will be quickly cleared from the body, so that the probes cannot reach the effective concentration at the lesion site. Compared with larger materials, nanoprobes exhibit the ability to enter VASPs due to the EPR effect. Moreover, they can be easily engineered to perform specific desirable functionalities such as active targeting, stimuli‐responsive imaging, extended circulation time by different surface modifications.^27^ These characteristics encourage researchers to design advanced nanoprobes that can outperform traditional modalities. Notably, rational design of the physicochemical properties of nanoprobes may benefit their targeting efficiency and biocompatibility.

Surface functionalization: Intravenous injection is the most effective route of administration. After intravenous administration, biomolecules (such as opsonin) in the blood will be labeled on the surface of the nanoprobes to form a “corona”, which is then recognized by the phagocytic system of reticuloendothelial cells (RES), resulting in the clearance of nanoprobes.^71^ To prolong the systemic circulation of nanoprobes and increase their accumulation in VASPs, nanoprobes are usually designed to be coated with polymers (such as polyethylene glycol (PEG)^72^ and hyaluronic acid (HA)^14^) or biomimetic membranes (such as red blood cell (RBC) membranes, leukocyte membrane, macrophage membranes, macrophage‐derived exosomes, platelet membrane and platelet‐derived extracellular vesicles^73^, ^74^). In particular, the biomimetic nanoprobes could bind specifically to certain biological components within the VASPs, further improving their targeting efficiency. In addition, some natural nanoparticles, such as ferritin nanocages,^20^, ^75^ high‐density lipoprotein^76^ and heat shock protein,^77^ could evade attack by the immune system and is therefore widely used for delivery of contrast agents.

Size: Except for the surface functionalization, the size of the nanoprobes themselves also play a huge role in their accumulation in VASPs. Tang et al. found that nanoparticles with hydrodynamic diameters of 7–30 nm exhibited a better in vivo pharmacokinetic behavior, showing longer half‐lives (5.0–6.3 h) compared with larger nanoparticles. In contrast, the plasma half‐life of nanoparticles with a diameter of 70 nm is only 0.67 h.^78^ This is because, when the nanoparticles are large, they are easily recognized by the immune system and thus cleared from the blood. When nanoparticles are too small (<6 nm), they are easily filtered out by the kidneys, leading to faster renal excretion.^79^ We hypothesize that the appropriate size of nanoparticles should be as small as possible given that it is bigger than 6 nm.

Shape: What is more, shape is also an important physicochemical property in the rational design of nanoprobes. It has been reported that the shape of nanomaterials such as spheres, rods, discs, and wires significantly affects the in vivo pharmacokinetic behavior of nanoparticles, their interactions with cells, and thus their targeting efficiency.^80^, ^81^, ^82^ Compared with spherical nanoparticles, non‐spherical nanoparticles exhibit superior performance in cellular uptake despite their more complex preparation method. This is due to the fact that non‐spherical nanoparticles have a large aspect ratio, which increases their contact area with the cells and promotes cellular uptake. While the curvature of the spherical nanoparticles makes them much less accessible to the vascular endothelial cells, thus reducing adhesion.^83^ But it is also for this reason that the blood circulation time of non‐spherical nanoparticles is shorter as they are easily ingested by immune cells and thus cleared. Therefore, the cellular uptake efficiency and blood circulation time should be considered together when designing nanoprobes.

Overall, nanoprobes, due to their unique size effect and easy modification, can ensure long circulation properties in the bloodstream and thus target accumulation to plaque sites. When designing a nanoprobe, we should make its particle size larger than 6 nm as much as possible, but not too much so that they can be better targeted to the lesion site through the EPR effect. In addition, modifying the nanoprobes with targeting moieties that can recognize the plaque features can enable them to accumulate to the plaque site and improve the imaging contrast, and then visualize the VASPs.

## 5 NANOPROBES FOR THE FEATURES OF VASPs VISUALIZATION

To achieve specific visualization of early VASPs features, numerous nanoprobes have been designed to target the biomarkers related to these features. Next, we briefly review the biomarkers related to these features and the corresponding nanoprobes (Table 2).

**TABLE 2:** Summary of the biomarkers, nanoprobes, targeting moieties and imaging modality mentioned in this article.

Features	Biomarkers	Nanoprobes	Targeting moieties	Imaging modality	Ref
Lipid‐rich necrotic core (lipids, apoptotic cells)	Lipids	RBC/LFP@PMMP	LFP	FI	84
	PS	AnxA5‐micelles	Annexin A5	FI/MRI	85
	CD47	Anti‐CD47 NP	Anti‐CD47 antibody	FI	86
Thin fibrous cap	MMP‐2	AuNRs‐Abs	MMP‐2 antibodies	PA	87
		MMP2cNPs	MMP‐2‐cleavable peptide	PET/MRI	88
	CTB	P‐ICG2‐PS‐Lip	KGGGFLGK peptide	FI	89
Inflammation	SR‐A	Fe‐PFH‐PLGA/CS‐DS	DS	MRI	90
	CD44	Cy5.5‐labeled HA‐NPs	HA	FI	91
	CD36	UCNPs@SiO2‐CD36	Anti‐CD36 antibody	FI	92
	MACRO	Anti‐MARCO UCNPs	Anti‐MACRO antibody	FI/MRI	93
	FR	^125^I/^131^I−Pd@Au‐PEG‐FA	FA	SPECT/CT/PA	94
	MPO	5HFeC NPs	5‐HT	MPI	
Neo‐angiogenesis	αvβ3	IONP‐cRGD‐NC	cRGD	MRI	95
	VEGFR	VEGFR‐2‐targeted NC	Anti‐VEGFR‐2 antibodies	US/MRI	96
	Unclear	68Ga‐NGD‐MNPs	GEBP11 peptide	PET/MRI	97
Microcalcification	Calcium	CC‐Aln	Aln	MRI	98
Multiple features	Lipid/CTB	L‐CRP	Alkyl chain/CTB‐responsive dipeptide	PA	99

### 5.1 Nanoprobes for lipid‐rich necrotic core visualization

**FIGURE 2:**
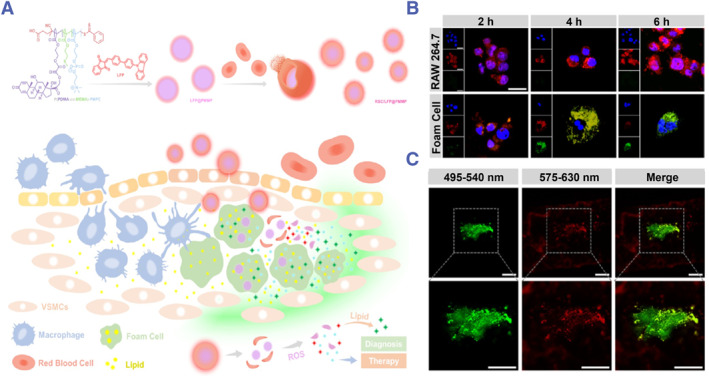
(A) Schematic diagram of the construction and mechanism of RBC/LFP@PMMP. (B) Confocal images of RAW 264.7 and foam cells treated with RBC/LFP@PMMP. (C) Confocal images of *en face* LFP‐stained aortas. The scale bar was 25 μm. Reproduced with permission.^84^ Copyright 2021, American Chemical Society.

Expansion of the necrotic core is the direct cause of VASPs rupture. Enlargement of the necrotic core, formed by the accumulation of lipid and apoptotic cells in the intima, irreversibly disrupts tight tissue. To enable lipid‐specific visualization in VASPs, Ma et al. designed a lipid‐specific fluorophore (LFP) that produces strong emission at 580 nm with orange fluorescence in water, while produces strong emission at 525 nm with green fluorescence in oil.^84^ Then, the authors packed LFP with oxygen species (ROS)‐responsive micelles, and the micelles were further coated with RBC membrane to obtain biomimetic nanoparticles (RBC/LFP@PMMP). After intravenous injection, it accumulates in plaques and releases LFP under the stimuli of ROS. Then, LFP combined with lipids in plaques and showed obvious green fluorescence (Figure 2A). As shown in Figure 2B, RBC/LFP@PMMP emits orange fluorescence in RAW 264.7 (normal macrophages) and green fluorescence in foam cells (lipid‐rich macrophages). Next, the authors investigated the ability of LFP to recognize VASPs in vivo. As shown in Figure 2C, LFP in plaques emitted a distinct green fluorescence, whereas LFP remaining in the arterial wall emitted an orange fluorescence. This work not only provides a lipid‐specific visualization strategy but also a nanoscale delivery system that responds to endogenous stimuli to accurately modulate drug release.

**FIGURE 3:**
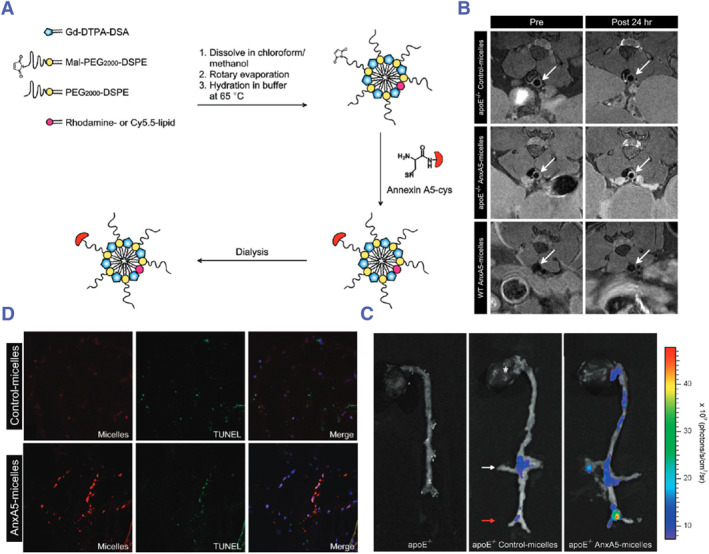
(A) Schematic diagram of the construction of AnxA5‐micelles. (B) T_1_‐weighted in vivo MR images at the level of the abdominal aorta (arrow). (C) Ex vivo near‐infrared fluorescence imaging of whole aortas. (D) Immunofluorescence section from abdominal aortas 24 h after administration. Reproduced with permission.^85^ Copyright 2010, American Chemical Society.

The apoptotic cells are another major component of the necrotic core. The apoptosis of macrophages and smooth muscle cells that have phagocytosed lipids leaves too many residues in the lesion, which eventually aggravates the expansion of the necrotic core. Therefore, the apoptotic cells were considered as important diagnostic and therapeutic targets for atherosclerosis. These apoptotic cells are characterized by exposure of phosphatidylserine (PS) and CD47 on their surface.^100^ For example, Nicolay et al. developed an annexin A5‐modified (a targeting ligand for PS) micellar nanoparticle (AnxA5‐micelles) as shown in Figure 3A, composed of Gd‐labeled lipids for MRI and Cy5.5‐lipid for fluorescence imaging (FI).^85^ At 24 h post‐administration of micelles, the T_1_‐weight signal of the ApoE^−/−^ group (VASPs mice) was significantly enhanced, while there was no significant change in WT group (normal mice). Furthermore, ApoE^−/−^ mice that were subjected to control‐micelles (no annexin A5‐modified) exhibited less signal enhancement (Figure 3B). Fluorescence imaging also showed similar results (Figure 3C). Next, the author performed TUNEL analysis on the plaques and found that compared with the control micelles, the fluorescence of the plaques treated with AnxA5‐micelles significantly increased. In addition, the fluorescence of AnxA5‐micelles almost completely overlapped with apoptotic cells (Figure 3D), indicating that AnxA5‐micelles are highly targeted to apoptotic cells. This study demonstrates the potential of annexin A5‐modified nanoprobes for target visualization of apoptotic cells in VASPs.

**FIGURE 4:**
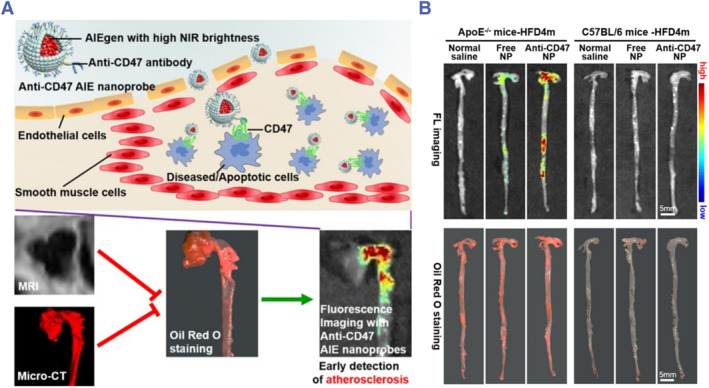
(A) Schematic diagram of the structure and mechanism of Anti‐CD47 NP. (B) Representative FL images and Oil Red O staining images of the whole aorta isolated from ApoE^−/−^ mice‐HFD4m and C57BL/6 mice‐HFD4m. Reproduced with permission.^86^ Copyright 2022, Wiley‐VCH.

CD47 is known to produce a “don't eat me” signal, thereby preventing macrophages from phagocytosis of tumors.^101^ Recently, an emerging study has shown that CD47 is also overexpressed on the surface of apoptotic cells within plaques to prevent macrophages from clearing apoptotic cells and promote the development of necrotic core.^102^, ^103^ Therefore, Ding et al. constructed an Anti‐CD47 antibody‐modified AIE nanoprobe for the early detection of VASPs.^86^ As shown in Figure 4A, the Anti‐CD47 NP can enter the plaque site through the broken vascular endothelium and binds specifically to CD47 on the surface of apoptotic cells. Anti‐CD47 NP showed significantly more fluorescence intensity at the plaque site than the unmodified probe (Free NP). In addition, in the C57BL/6 mice (healthy mice), the fluorescence intensity was significantly lower, indicating that the specificity of the Anti‐CD47 NP for VASPs (Figure 4B). This work enabled high contrast and accurate early detection of VASPs, which was not detectable by either clinically used micro‐CT or MRI and demonstrated the potential of CD47 as a target to detect VASPs.

### 5.2 Nanoprobes for thin fibrous cap visualization

The fibrous cap acts as a plaque barrier, and its thinning is closely related to plaque rupture. As mentioned above, proteolytic enzymes, such as matrix metalloproteinases (MMPs) and cysteine proteases, could degrade extracellular matrix to increase the instability of plaque. Thus, the elevated expression of protease has laid the foundation for developing enzyme‐targeting probes for accurately visualizing VASPs.^104^

**FIGURE 5:**
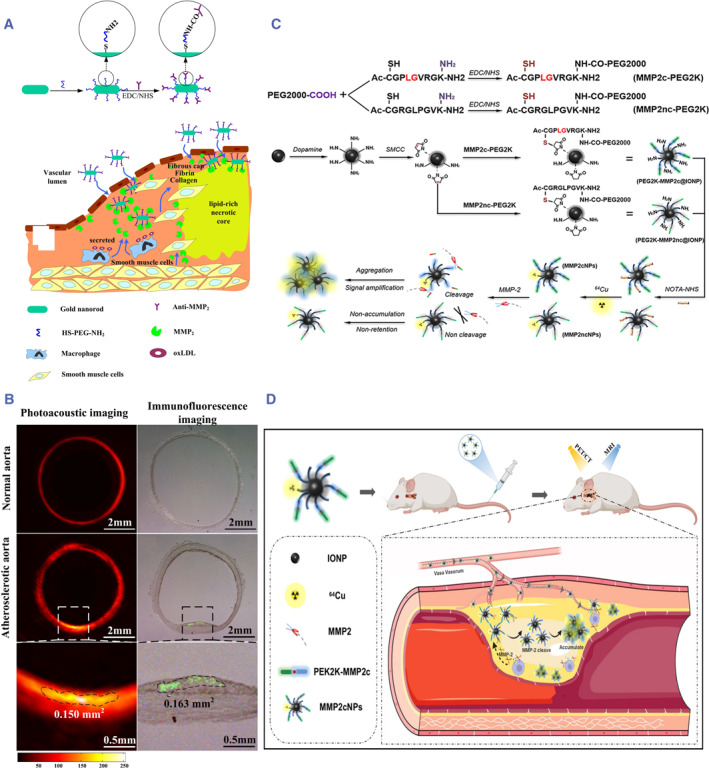
(A) Schematic diagram of the construction and action mechanism of AuNRs‐Abs. (B) Photoacoustic imaging and immunofluorescence imaging of normal and atherosclerotic aorta after AuNRs‐Abs injection. Reproduced with permission.^87^ Copyright 2016, Elsevier. (C) Schematic illustration of the preparation of MMP2cNPs and the non‐cleavable reference (MMP2ncNPs). (D) Schematic diagram of the mechanism of MMP2cNPs. Reproduced with permission.^88^ Copyright 2022, The Authors, published by Dove Medical Press Limited.

MMPs are a family of enzymes with zinc ions as the catalytic center that degrade the components of the extracellular matrix (ECM) in atherosclerotic plaques. Among them, matrix metallopeptidase‐2 (MMP‐2) is emerging as an important biomarker of VASPs.^105^ Xing et al. fabricated a photoacoustic (PA) imaging probe (AuNRs‐Abs) that conjugated MMP‐2 antibodies with gold nanorods to localize MMP‐2 for identification of VASPs (Figure 5A).^87^ Gold nanorods exhibit strong light absorption capabilities in the near‐infrared region and have been extensively studied as an attractive photoacoustic contrast agent due to their high biocompatibility. As shown in Figure 5B, after being modified by MMP‐2 antibodies through the EDC/NHS condensation reaction, it can specifically bind to the highly expressed MMP‐2 in plaques and enhance photoacoustic signals compared with non‐targeted gold nanorods (AuNRs‐PEG). Interestingly, this study not only visualized the plaque morphology with the assistance of enhanced photoacoustic signals but also revealed the distribution of MMP‐2 within plaques and provided a quantitative assessment.

In another study, Cheng et al. synthesized MMP‐2‐cleaveable nanoprobes to amplify the PET/MRI signal in VASPs.^88^ Specifically, PEG_2k_ was modified with MMP‐2‐cleavable peptide to obtain MMP2c‐PEG_2k_ and linked with iron oxide, and then labeled with Cu^64^ to obtain intelligent MMP2cNPs (Figure 5C). Under the stimulation of MMP‐2 in VASPs, the MMP2cNPs will gather and amplify the PET/MRI signal to detect VASPs (Figure 5D). In contrast, the PET/MRI signal of non‐cleavable probes exhibited less accumulation in VASPs, so the PET/MRI signal was lower. This study demonstrated the feasibility of MMP‐2‐responsive PET/MEI nanoprobes for the identification of VASPs.

**FIGURE 6:**
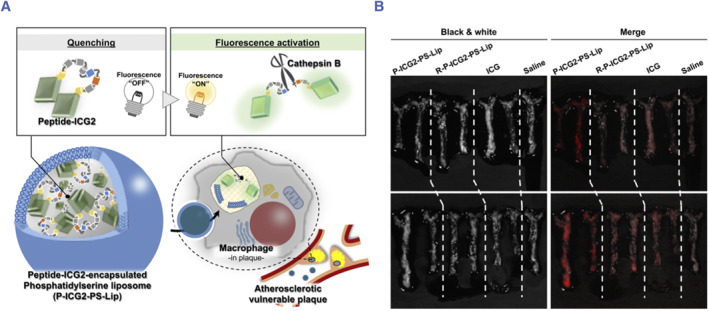
(A) Schematic illustration of the mechanism of P‐ICG2‐PS‐Lip for NIRF imaging of VASPs. (B) Fluorescence images of aortas from different groups. Reproduced with permission.^89^ Copyright 2019, Elsevier.

Cathepsin B (CTB) is a typical protease secreted by macrophages. Abnormal elevation of its activity can break the structural integrity of plaques, exposing the internal components of the plaque to the blood, leading to thrombus. Numerous studies have confirmed the role of CTB in vasculopathy processes.^106^, ^107^ Therefore, CTB is an attractive biomarker for assessing plaque vulnerability. For example, Ogawa et al. synthesized a CTB‐targeting fluorescent probe (Peptide‐ICG2) for the detection of VASPs.^89^ As shown in Figure 6A, peptide‐ICG2 was synthesized by coupling two indocyanine green (ICG) with a linker peptide (KGGGFLGK) containing the CTB‐cleavable sequence. The fluorescence of Peptide‐ICG2 was quenched until cleaved by CTB. To improve the targeting of the probe to the plaque, the authors encapsulated the probe in liposomes containing PS, named P‐ICG2‐PS‐Lip, so that it can bind to macrophages that are abundant in the plaque. In vivo study shows that the fluorescence signal of ICG is strongest in the aorta of mice treated with P‐ICG2‐PS‐Lip, compared with ICG and non‐specific R‐P‐ICG2‐PS‐Lip (Figure 6B). Thus, enzyme activity‐responsive probes can reduce nonspecific noise, thereby improving the signal‐to‐noise ratio for targeting lesions and accurately displaying plaque vulnerability.

### 5.3 Nanoprobes for inflammation visualization

**FIGURE 7:**
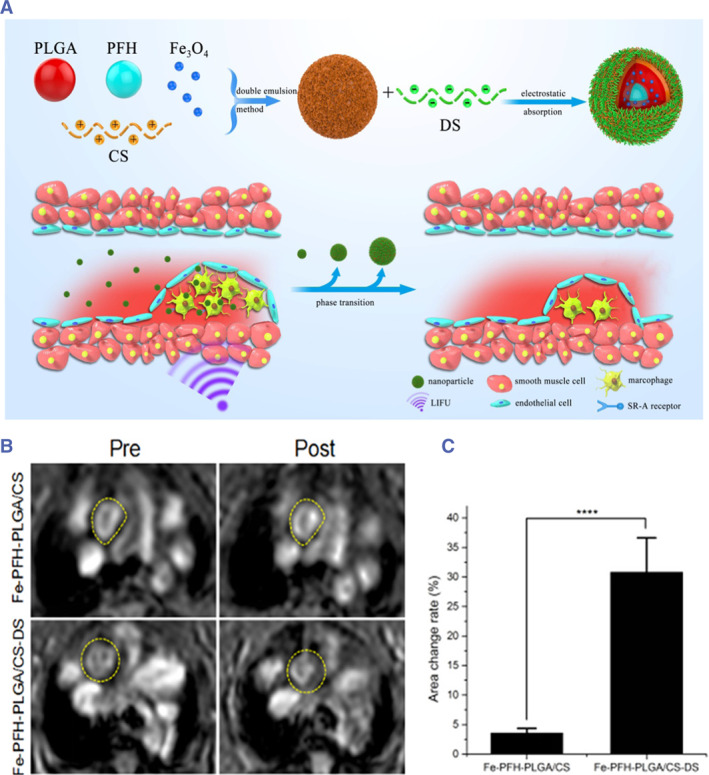
(A) Schematic diagram of the preparation and mechanism of the Fe‐PFH‐PLGA/CS‐DS. (B) T_2_‐weighted MRI of before and 2 h after the nanoprobes injection. (C) The area change rate of the Fe‐PFH‐PLGA/CS and Fe‐PFHPLGA/CS‐DS groups. Reproduced with permission.^90^ Copyright 2019, American Chemical Society.

Inflammation is present throughout AS and is closely related to plaque vulnerability. The pathological process of VASPs involves the participation of a variety of inflammatory cells and pro‐inflammatory mediators. Among them, macrophages are the most important inflammatory cells that contribute to plaque vulnerability and are most abundant in VASPs sites.^108^ Macrophage levels in VASPs have been reported to be as much as three to five times higher than in stable plaques, making macrophage aggregation an important indicator of plaque vulnerability.^25^ It aggravates the development of plaques by releasing a variety of inflammatory mediators such as interleukin‐6 (IL‐6), interleukin‐1 (IL‐1), and MCP‐1, and at the same time secretes proteolytic enzymes to thin the plaque fibrous cap.^109^ Macrophages within vulnerable plaques often express high levels of multiple receptors, such as scavenger receptor A (SR‐A), CD44, CD36, a collagenous structure (MARCO), folate receptors (FR), and so on.^110^, ^111^, ^112^ Researchers often use corresponding ligands or antibodies to couple with nanoprobes to actively bind macrophages to achieve specific imaging of VASPs. For example, Guo et al. synthesized an SR‐A‐targeted nanoprobe (Fe‐PFH‐PLGA/CS‐DS) by encapsulating Fe_3_O_4_ and perfluorohexane (PFH) with PLGA and modifying chitosan (CS) on the surface, which in turn adsorbed dextran sulfate (DS) through electrostatic interaction (Figure 7A).^90^ DS is a ligand for SR‐A, enabling Fe‐PFH‐PLGA/CS‐DS to actively target inflammatory macrophages. As a result, at 2 h of probe injection, MRI signals in the aorta of Fe‐PFH‐PLGA/CS‐DS‐treated mice were significantly attenuated (Figure 7B), with an area change rate as high as 30.80% (Figure 7C). Thus, the SR‐A targeting nanoprobes achieve more accumulation in VASPs, leading to superior imaging performance. In addition to nanoprobes targeting SR‐A, there are nanoprobes targeting CD44 (Cy5.5‐labeled HA‐NPs),^91^ CD36 (UCNPs@SiO_2_‐CD36),^92^ MACRO (anti‐MARCO UCNPs),^93^ and FR (^125^I/^131^I−Pd@Au‐PEG‐FA)^94^ to recognize macrophages for the purpose of detection of VASPs.

**FIGURE 8:**
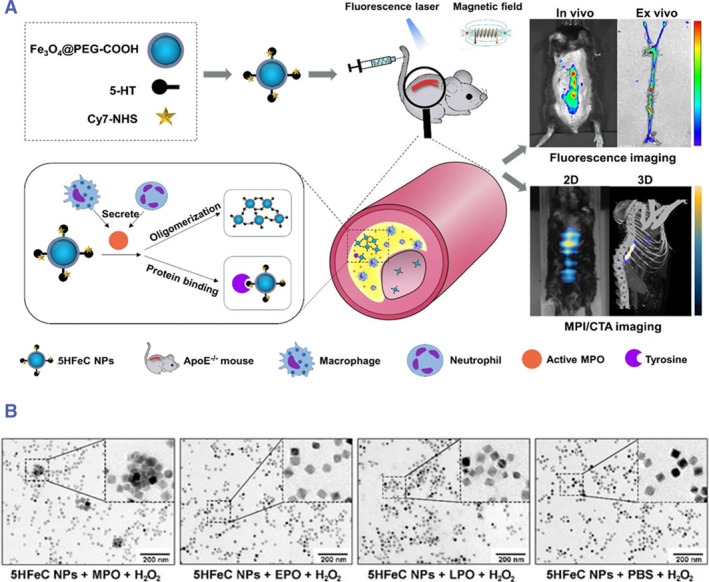
(A) Schematic illustration of the mechanism of 5HFeC NPs for MPI of VASPs. (B) TEM images of 5HFeC NPs after incubation with H_2_O_2_ and MPO, erythropoietin protein (EPO), lactoperoxidase (LPO), or PBS. Reproduced under terms of the CC‐BY license.^116^ Copyright 2021, The Authors, published by Ivyspring International Publisher.

Myeloperoxidase (MPO) is an inflammatory protein secreted by inflammatory cells that contributes to plaque vulnerability through several mechanisms, such as promoting the production of reactive oxygen species, depleting levels of anti‐inflammatory molecules and activating MMP.^113^ It has been shown that MPO is abundantly present in ruptured coronary plaques in patients who die of acute myocardial infarction.^114^ Recent animal studies have also indicated that the inhibition of MPO activity can stable VASPs.^115^ Thus, MPO is considered a potential inflammatory marker for identifying vulnerable atherosclerotic plaques. Chen et al. coupled 5‐hydroxytryptamine (5‐HT) and Fe_3_O_4_@PEG‐COOH with Cy7‐NHS to design a novel multimodal imaging nanoprobes (5HFeC NPs) for active MPO mapping during inflammation to specifically identify VASPs (Figure 8A).^116^ 5‐HT can self‐oligomerize or bind proteins under the catalyzation of active MPO in inflamed tissues, thus accumulating within plaques (Figure 8B). The in vivo FI and magnetic particle imaging results showed that the signal intensity of the probe was significantly stronger in vulnerable plaque model mice than that in healthy mice. This study revealed that active MPO‐monitored nanoprobes could serve as a strategy for the detection of VASPs.

### 5.4 Nanoprobes for neo‐angiogenesis visualization

**FIGURE 9:**
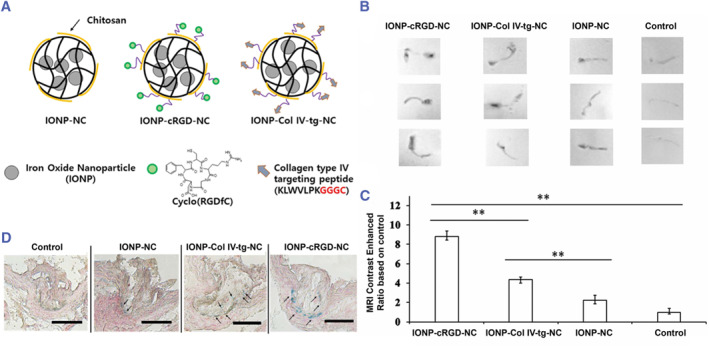
(A) Schematic illustration of the preparation of IONP‐NC, IONP‐cRGD‐NC and INOP‐Col IV‐tg‐NC. (B) T2‐weighted MR images and (C) MR contrast enhancement of isolated aorta from ApoE^−/−^ mice after injection of nanoprobes. (D) Prussian blue staining images of atherosclerotic plaque. Reproduced with permission.^95^ Copyright 2018, Elsevier.

In response to stimuli such as inflammation and hypoxia, neo‐vessels originate from the adventitial area and extend to the base of the plaque. These defective blood vessels are prone to rupture and leakage of inflammatory cells, eventually leading to hemorrhage and inflammation. Clinical studies have shown that the risk of plaque rupture is increased when neo‐angiogenesis occurs.^117^ Therefore, neo‐angiogenesis was regarded as a potential target for assessing VASPs. At present, the best popular markers involved in neo‐angiogenesis are integrin (αvβ_3)_ and vascular endothelial growth factor receptor (VEGFR).^118^ For example, Tae et al. constructed cRGD peptide‐modified chitosan‐containing Pluronic nanocarriers loaded with iron oxide nanoparticle (IONP‐cRGD‐NC) for systemic investigation of their targeting ability towards neo‐angiogenesis in VASPs.^95^ Moreover, they constructed collagen type IV targeting peptide‐modified nanocarriers (INOP‐Col IV‐tg‐NC) to compare the binding affinity of cRGD peptide with collagen type IV‐targeting peptide for VASPs (Figure 9A). The contrast signals in the aorta of mice treated with IONP loaded nano‐carries were all enhanced. Compared with the unmodified IONP‐NC, IONP‐cRGD‐NC exhibited about 4‐fold higher contrast enhancement, while INOP‐Col IV‐tg‐NC exhibited near 2‐fold higher contrast enhancement (Figure 9B,C). Prussian blue staining also demonstrated that IONP‐cRGD‐NC accumulated most at the VASPs, with INOP‐Col IV‐tg‐NC only second and IONP‐NC least (Figure 9D). They concluded that the cRGD peptide can be considered as a more suitable targeting ligand for nanocarrier systems than the collagen type IV‐targeting peptide at relatively early stages of VASPs. In another study, Wei et al. constructed anti‐VEGFR‐2 antibodies‐modified nano‐capsules, containing iron oxide nanoparticle for MRI and perfluorocarbon for US imaging to target visualizing neo‐angiogenesis.^96^ These results suggest that targeting receptors highly expressed in neo‐angiogenesis can increase imaging contrast at the lesion site and assess plaque vulnerability.

**FIGURE 10:**
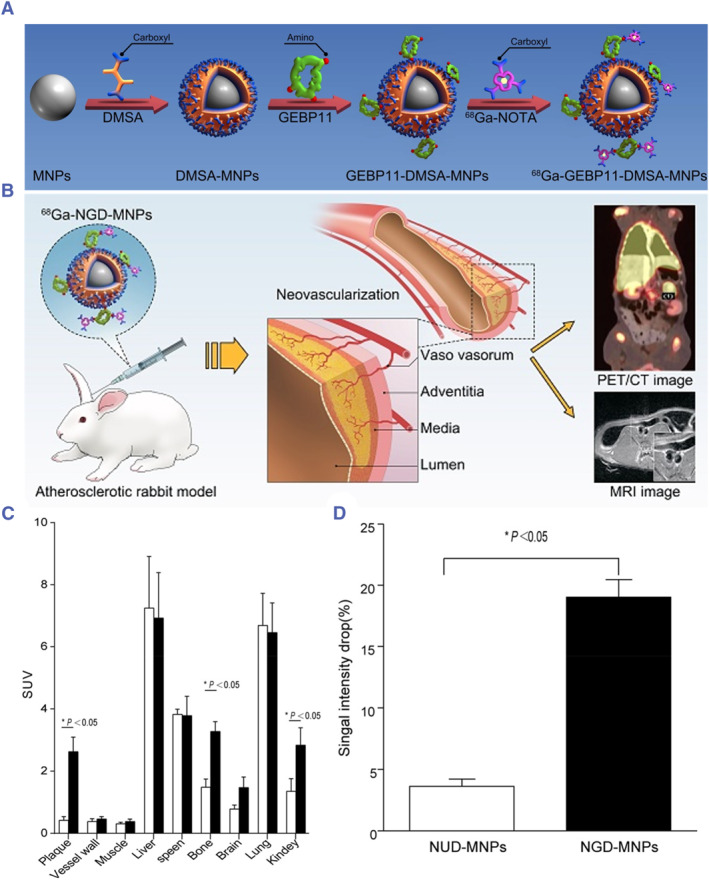
(A) Schematic illustration of the preparation of ^68^Ga‐NGD‐MNPs. (B) Schematic illustration of the application of 68Ga‐NGD‐MNPs for plaque neo‐angiogenesis molecular imaging with PET/MR. (C) Quantification of PET images. Plaque in ^68^Ga‐NGD‐MNPs group exhibited significantly greater mean standardized uptake value (SUV) than control group. (D) Comparison of the relative signal intensity change (NENH%) in the T2‐weighted images of abdominal arterial wall before and 4 h after NGD‐MNPs or NUD‐MNPs injection. Reproduced with permission.^97^ Copyright 2017, Ivyspring International Publisher.

In addition to these two targets, Cao et al. recently identified a new molecule capable of targeting and recognizing neo‐angiogenesis, GEBP11 peptide, a cyclic peptide composed of nine amino acids (CTKNSYLMC).^97^ It performs high affinity and specificity for neo‐angiogenesis of vascular endothelial cells. As shown in Figure 10A, they conjugated it to Fe_3_O_4_ magnetic nanoparticles and radiolabeled with ^68^Ga to obtain ^68^Ga‐NGD‐MNPs. Sixty minutes after the probe was injected into anesthetized rabbits via a marginal ear vein, PET clearly visualized the accumulation of ^68^Ga‐NGD‐MNPs in the aorta compared with the control group (^68^Ga‐NUD‐MNPs). MRI also showed that ^68^Ga‐NGD‐MNPs effectively shortened the T_2_ relaxation time and produced a significant negative enhancement of T_2_‐weighted images compared with ^68^Ga‐NUD‐MNPs (Figure 10B–D). This study developed a novel PET/MR dual‐mode imaging probes (NGD‐MNPs) with high specificity using self‐identified targeting molecules, which provide a new strategy for non‐invasive assessment of angiogenesis in VASPs. However, the receptor for GEBP11 peptide in neo‐angiogenesis is still unclear and needs to be further explored in detail.

### 5.5 Nanoprobes for microcalcification visualization

**FIGURE 11:**
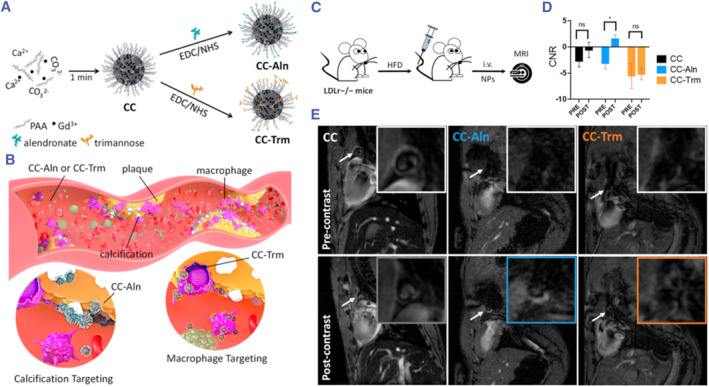
(A) Schematic illustration of the preparation of CC‐Aln and CC‐Trm. (B) Illustration of the mechanism of CC‐Aln and CC‐Trm in atherosclerotic plaques. (C) Schematic of targeted MRI of atherosclerosis in LDLr^−/−^ mice with nanoprobes. (D) Comparison of CNR enhancement of the aortic arch tissue with targeted CC NPs (CC vs. CC‐Aln and CC‐Trm NPs). (E) T_1_‐weighted MRI before (pre‐contrast) and after 1 h (post‐contrast) of the injection of nanoprobes. Reproduced with permission.^98^ Copyright 2023, American Chemical Society.

Emerging evidence indicates that intimal calcification is a healing response to serious inflammation and cell death within VASPs.^119^, ^120^ Calcium deposits increase the volume of the necrotic core of advanced plaques on the one hand, and on the other hand, they increase the blood flow shear stress on the plaque, leading to plaque rupture. It has been reported that microcalcification is a key hallmark of VASPs, confirming its important role in elevating cardiovascular risk.^121^, ^122^ Carregal‐Romero et al. fabricated amorphous calcium carbonate nanoparticles doped with gadolinium (CC) for MRI.^98^ To target microcalcification, they obtained CC‐Aln by modifying CC with alendronate through the EDC/NHS method (Figure 11A). Moreover, they compared CC‐Aln with trimannose‐modified CC (CC‐Trm) that targets inflammation (Figure 11B). LDLr^−/−^ mice fed a high‐fat diet were used as the model. Sixty minutes after tail vein injection, CC‐Aln accumulated in large amounts in the VASPs, demonstrating a bright T_1_ signal and significant increased contrast‐to‐noise ratio (CNR). In contrast, mice subjected to CC and CC‐Trm showed little bright T_1_ signal, with no significant difference in CNR from pre‐injection (Figure 11C–E). This work not only constructs nanoprobes that can specifically visualize the microcalcification phenomenon and thus identify the detection of VASPs, but also compares the ability of different ligand‐modified nanoprobes to accumulate to the VASPs, which provides a new idea for the future construction of nanoprobes with stronger binding affinity to VASPs.

### 5.6 Nanoprobes for multiple features visualization

**FIGURE 12:**
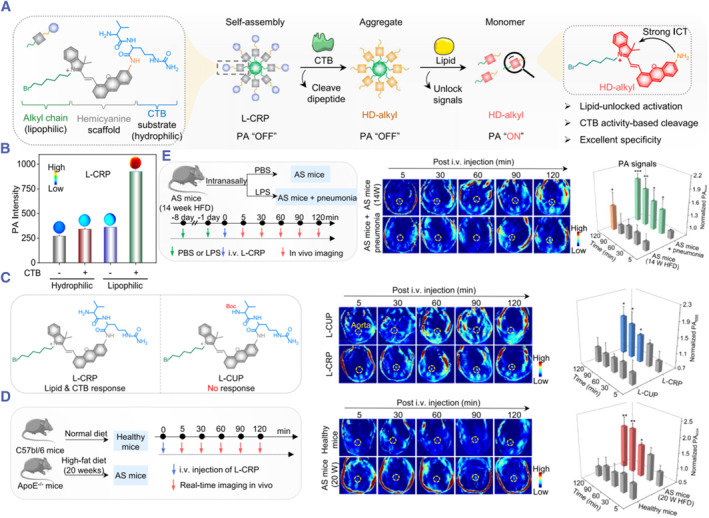
(A) Schematic illustration of Chemical structure and the responsive progress of lipid‐unlocked CTB responsive probe (L‐CRP). (B) PA intensity of L‐CRP at 695 nm after incubation with CTB (0 or 75 U/L) in hydrophilic (CTB working buffer) or lipophilic environments (CTB working buffer containing 33% EtOH). (C) PA images and intensity of AS mice after injection with LCRP or L‐CUP over time. (D) PA images of healthy and AS mice after injection of L‐CRP over time. Yellow dotted curves indicate aortic regions. (E) PA images and intensity of AS mice and AS mice + pneumonia after injection of L‐CRP over time. Reproduced with permission.^99^ Copyright 2023, American Chemical Society.

Due to the complexity of human physiology, pathological markers of VASPs are sometimes expressed in normal tissues, resulting in compromised specificity. The plaque environment is so complex that the detection of a single marker often does not provide sufficiently valid information for a comprehensive assessment of the disease and may lead to misdiagnosis. Therefore, simultaneous detection of multiple pathological features is important for improving diagnostic accuracy. Given the critical roles of lipids and CTB in vulnerable plaques, Zhang et al. designed a lipid‐unlocked CTB‐activated photoacoustic probe (L‐CRP) by linking a CTB‐responsive hydrophilic dipeptide, a lipophilic alkyl chain, and a hemicyanine dye (Figure 12A).^99^ L‐CRP exhibits little PA signals in its locked state, whereas its PA signals are only activated in the presence of CTB and lipids (Figure 12B). For in vivo imaging experiments, they synthesized a non‐sensitive probe (L‐CUP) as a control. The L‐CRP group exhibited significantly enhanced PA signals in the aortic region within 120 min. In contrast, there was no significant change in PA signals in the L‐CUP group (Figure 12C). These results suggest that the elevation of PA signal in the aortic region was due to the activation of CTB by L‐CRP. Furthermore, L‐CRP could specifically identify atherosclerotic mice (Figure 12D). Moreover, there was a significant difference in PA signals between AS mice and AS mice with pneumonia, suggesting its potential for high‐risk stratification of AS mice (Figure 12E). This study constructed a nanoprobe that releases PA signals only when multiple features of VASPs are present, increasing the accuracy of VASPs detection and providing new ideas for future construction of nanoprobes with high accuracy.

## 6 SUMMARY AND PERSPECTIVES

In this review, we elaborate on the pathology of AS, the features of VASPs, current clinical imaging technologies, rational design of nanoprobes and their application in the visualization of VASPs features, with a view to providing references for the better development of the field. The nanoprobes can be rationally modified to image biomarkers that are closely related to the features of VASPs, improving imaging contrast and assessing plaque vulnerability. In particular, the nanoprobes that can simultaneously detect multiple features improve diagnostic accuracy, promising to help clinicians better grade the risk of VASPs.

In the past decades, there has been a breakthrough in the application of nanoprobes in cardiovascular diseases, which has become the second most important branch of application after cancer. Nanoprobe‐based molecular imaging techniques allow non‐invasive characterization of plaque composition. The development of advanced molecular imaging nanoprobes will provide versatile tools for target‐specific imaging of atherosclerotic bioprocesses, including inflammation, lipid‐rich necrotic core, thin fibrous caps, microcalcifications, and neo‐angiogenesis. In addition, emerging nanoprobes that can be customized to display multiple features accurately have been developed for improved accuracy in the diagnosis of VASPs. Moreover, nanoprobes could be modified with various targeting moieties to accumulate at the lesion site, thereby improving imaging contrast at the lesion site. These engineered probes help to accurately distinguish atherosclerotic diseased tissue from normal tissues. In the context of the era of precision medicine, these nanoprobe‐based molecular imaging will make outstanding contributions in improving patient care, disease management, and research for a deeper understanding of disease mechanisms. Although still in the research stage, they show attractive prospects in the early detection of VASPs. However, there are still several key challenges and issues that need to be urgently addressed to achieve clinical translation and long‐term development:

1. **Biosafety**. The accumulation of nanoprobes in human tissues could trigger certain toxicity, especially when the particle size of nanoprobes is small enough, there is a possibility to cross the blood‐brain barrier and accumulate in the brain, so it is necessary to evaluate the acute toxicity of nanoprobes. In fact, acute toxicity evaluation of nanoprobes is not sufficient to evaluate their biosafety for many reasons, and chronic toxicity of nanoprobes is also an important factor to consider. For example, patients who need to be given nanoprobes for a long period of time will accumulate a certain concentration of nanoprobes in their bodies; therefore, the distribution of the probes in the body needs to be examined and the safety of the organ evaluated. In addition to patients, staff members need to be exposed to the nanoprobes for years and years, so the chronic toxicity of the probes needs to be evaluated in detail.^123^
2. **Specificity**. The nanoprobes in current research are constructed based on the biomarkers provided by the pathological features of VASPs, and diagnosis is achieved by imaging the biomarkers of the VASPs. However, most biomarkers are not specific and may be highly expressed in other diseases making current imaging strategies controversial.^13^ Therefore, a profound understanding of the pathology of VASPs and the search for specific biomarkers is crucial. In addition to this approach, simultaneous imaging of multiple features is also a promising strategy as the likelihood of multiple biomarkers being present at the same time in other diseases is negligible.^124^
3. **Targeting Efficiency**. Effective accumulation of nanoprobes in VASPs is a prerequisite for improving imaging performance. Strategies for nanoprobes accumulation at VASPs are categorized into passive targeting and active targeting, both based on the EPR effect; however, there has been an ongoing controversy about the EPR effect because many researchers believe that the EPR effect does not exist in human models.^17^, ^125^ Passive targeting exhibits low targeting efficiency, and although active targeting strategies can enhance targeting efficiency, they still suffer from low targeting efficiency and instability in complex living systems. The reason for this may be the low binding affinity of the ligand to the receptor, which cannot resist blood washout, resulting in a low concentration of the probe at VASPs.^126^ Therefore, there is an urgent need to optimize the targeting moieties of the nanoprobes to enhance its binding affinity to the target site. In addition, improving the stability of the nanoprobes also contributes to their accumulation at the target site.
4. **Reproducibility**. The reproducibility of nanoprobe performance is an important parameter for its industrialization. Although the nanoprobes showed homogeneous performance in laboratory preparations. However, scaling up to industrial‐scale production, challenges in terms of encapsulation rate, particle size and homogeneity have emerged.^25^ Current nanoprobes rely on complex preparation methods, leading to poorly reproducible properties, which seriously limits their industrialized production. Therefore, it is crucial to simplify the preparation methods of the nanoprobes.

Clinical translation of nanoprobes is a long and complex process that requires the combined efforts of materials scientists, pathologists, toxicologists, clinicians, and companies. Despite the long and thorny road, we believe that based on the many successes of nanoprobes in preclinical studies for the detection of VASPs and their application in cancer diagnosis and treatment, they will eventually be used for the diagnosis of VASPs.

## AUTHOR CONTRIBUTIONS

Bing Zhang and Xin Wang conceived the idea; Xin Wang and Dan Mu wrote the manuscript; Jing Liang, Ruijing Xin, Yukun Zhang, Renyuan Liu and Mei Yao revised the manuscript.

## CONFLICT OF INTEREST STATEMENT

The authors declare that there are no competing interests.
